# Pro- and anti-inflammatory cytokine dysregulation in the saliva of patients with acromegaly: a cross-sectional study

**DOI:** 10.1007/s11102-026-01767-6

**Published:** 2026-10-16

**Authors:** Renata Carvalho Campelo, Mila Roselaine Lima de Assunção, Jessilene Ribeiro Rocha, Luís Gustavo Souza Santos, Erika Sales Lopes, Sabrina da Silva Pereira Damianse, Rossana Santiago de Sousa Azulay, Manuel dos Santos Faria, Gilvan Cortês Nascimento, Vandilson Pinheiro Rodrigues

**Affiliations:** 1 Dentistry Graduate Program, Federal University of Maranhão, Avenida dos Portugueses, 1966, Bacanga, São Luís, Maranhão 65080-805 Brazil; 2 Research Group in Clinical and Molecular Endocrinology and Metabolism, Federal University of Maranhão, São Luís, Brazil; 3 Service of Endocrinology, University Hospital of the Federal University of Maranhão, São Luís, Brazil

**Keywords:** Acromegaly, Saliva, Cytokines, Inflammation

## Abstract

**Background:**

There is evidence to suggest that hormonal dysregulation induced by acromegaly may affect serum cytokine levels. However, to our knowledge, no previous studies have examined the salivary cytokine profile in adults with acromegaly. The aim of this study was to identify the salivary profile of pro- and anti-inflammatory cytokines associated with acromegaly in adults.

**Methods:**

A cross-sectional study was conducted with 25 patients with acromegaly and age-, 25 age- and sex-matched controls. Demographic and general health data were collected. Saliva samples were obtained to quantify the concentrations of IL-1β, IL-4, IL-6, IL-10, IL-17A, IL-22, TGF-α, and TNF-α. Statistical analyses included the Mann-Whitney test, Spearman's correlation, and ROC curve analysis.

**Results:**

Patients with acromegaly exhibited significantly higher salivary levels of IL-4, IL-6, IL-10, TGF-α, and TNF-α (p<0.001), as well as IL-17A (p=0.029). No significant differences were observed for isolated IL-1β and IL-22. Comparative analysis showed significantly lower IL-22/IL-4 (p<0.001), IL-1β/IL-10 (p=0.023), and IL-22/IL-10 (p=0.003) ratios in patients with acromegaly compared with controls. Given that IL-1β and IL-22 did not differ significantly between groups, whereas IL-4 and IL-10 were increased in acromegaly, these lower ratios appear to primarily reflect higher levels of the anti-inflammatory cytokines. In multivariate analysis, IL-4, IL-6, IL-17A, IL-22, and TNF-α remained statistically associated with acromegaly.

**Conclusion:**

The study findings suggest that patients with acromegaly exhibit a significant imbalance in the levels of pro- and anti-inflammatory cytokines in saliva, with emphasis on IL-4, IL-6, IL-17A, IL-22, and TNF-α.

**Supplementary Information:**

The online version contains supplementary material available at https://doi.org/10.1007/s11102-026-01767-6.

## Introduction

 Acromegaly is a rare disorder, typically caused by a benign tumor in the pituitary gland, leading to the excessive production of growth hormone (GH) and insulin-like growth factor-1 (IGF-1). Although the tumor is benign, acromegaly has a significant impact on overall health and is frequently associated with a wide range of metabolic, cardiovascular, respiratory, musculoskeletal, neurological, and neoplastic comorbidities [1–3]. Furthermore, it impairs quality of life, including oral health [4]. In the orofacial region, patients with acromegaly present with alterations such as mandibular prognathism, macroglossia [5, 6], increased salivary gland volume [7], and increased salivary flow [8].

These comorbidities may induce a state of systemic inflammation, characterized by elevated serum cytokine levels, which are fundamental mediators in both physiological and pathological immune responses. Depending on the context, cytokines can exert both beneficial and deleterious effects on the organism [9]. The measurement of these cytokines in various biomaterials, including saliva, has been investigated due to its diagnostic and prognostic relevance. Saliva shows a significant correlation with serum levels of these inflammatory molecules, making it a promising biological fluid for assessing the inflammatory status of patients [10–12].

Pro-inflammatory cytokines, such as interleukin (IL)-1β, IL-6, IL-8, and tumor necrosis factor (TNF)-α, among others, play a crucial role in inducing the inflammatory cascade, a physiological response of the immune system to tissue injury [13]. Moreover, the analysis of salivary biomarkers has become a valuable tool for both the diagnosis and monitoring of diseases, providing crucial data for early and preventive intervention. Saliva collection is a non-invasive, low-cost approach with good stability and ease of collection, making it ideal for clinical practice in various conditions, such as diabetes, vascular diseases, Sjögren’s syndrome, certain types of cancer, and viral infections [14–16].

Despite growing evidence of oral changes in acromegaly, such as increased salivary gland volume and other oral manifestations [17], few studies have investigated the salivary cytokine profile in this context. Given the oral alterations associated with acromegaly that can affect saliva quality and reflect inflammatory status, there has been increased interest in studying these biomarkers in the saliva of patients with acromegaly. Therefore, the aim of this study was to investigate the profile of pro- and anti-inflammatory cytokines in the saliva of acromegaly patients. The hypothesis was that these biomarkers are related to the disease’s inflammatory state. These results could help identify salivary molecules that aid in assessing disease activity, monitoring inflammatory status, and evaluating treatment response.

## Materials and methods

### Study design and sample

A cross-sectional study was conducted with an acromegaly group and a control group in the city of São Luís, Maranhão, Brazil. The project was initially submitted and approved by the Research Ethics Committee of the University Hospital of the Federal University of Maranhão (HUUFMA) (CAAE: 33124820.6.0000.5086). All participants were informed about the objectives and procedures involved in the study and, after full understanding, signed an informed consent form. Additionally, all participants received guidance on prevention, oral hygiene procedures, and treatment of oral diseases, and were referred, when necessary, for dental treatment at the Clinics of the Dentistry School of the Federal University of Maranhão.

The acromegaly group consisted of patients of both sexes, aged 18 years or older, with a confirmed diagnosis of acromegaly by the medical team in accordance with established guideline [18], and who were monitored at the Endocrinology Outpatient Clinic of HUUFMA. Patients who were hospitalized, pregnant women, individuals with a body mass index (BMI) below 18.5, current smokers or former smokers for less than 5 years, and those with clinical signs of infectious and/or inflammatory salivary gland conditions were excluded.

The control group comprised non-acromegalic individuals treated at the Clinical Disciplines of the Dentistry Course of the Federal University of Maranhão. Individuals of both sexes, aged 18 years or older, were included in this group. Individuals who had used antibiotic and/or anti-inflammatory medications in the last 3 months, who had diabetes, pregnant women, women on estrogen hormone replacement therapy, individuals with a BMI below 18.5, current smokers, or former smokers for less than 5 years, and those with clinical signs of infectious and/or inflammatory salivary gland conditions were excluded. To minimize research biases, the control group was matched with the case group, considering age (within ± 4 years) and gender (male or female) of the participants.

### General data collection

A semi-structured questionnaire was used to collect sociodemographic and self-reported health data from the participants. Participants’ weight (in kilograms) and height (in meters) were measured to calculate their body mass index (BMI in kg/m²). After completing the questionnaire, the patient’s medical records were consulted in the case group to collect medical data on the presence or absence of comorbidities such as arterial hypertension, diabetes, chronic kidney disease, arthrosis, and dyslipidemia, as well as serum data (fasting glucose, HbA1c, and leukocytes). IGF-1 levels were expressed as a percentage of the upper limit of normal (% ULN), based on age- and sex-specific normal ranges, if available. Biochemically controlled acromegaly was defined as an age- and sex-adjusted normal serum IGF-1 level [19].

### Saliva collection

Unstimulated saliva samples were collected. Patients were instructed not to eat or brush their teeth for 60 min before collection. During the test, the patient tilted their head forward and was instructed not to speak or swallow saliva, and then spat the volume into a 50 mL graduated Falcon-type collection tube. For saliva collection, participants were instructed to avoid swallowing their saliva for 5 min and to spit all collected saliva into a graduated receptacle every 60 s [8]. After collection, samples were frozen and stored at -40 °C until marker quantification.

### Salivary cytokine levels

Analyses of salivary levels (in pg/mL) of interleukin (IL)-1β, IL-4, IL-6, IL-10, IL-17 A, IL-22, transforming growth factor (TGF)-α, and tumor necrosis factor (TNF)-α (in pg/mL) were processed in duplicate using the multiplex methodology on the samples. The multiplex methodology is based on an immunoassay test that uses flow cytometry, in which capture antibodies specific for each analyte are linked to color-coded microspheres. A customized MILLIPLEX^®^ Human Cytokine/Chemokine/Growth Factor Panel A Magnetic Bead Panel (HCYTA-60 K, Merck Millipore, USA) was used for the simultaneous quantification of the eight molecules. The assays were conducted according to the manufacturer’s instructions.

### Statistical analysis

Data were analyzed using R software version 4.6.1 (R-Core Team) and GraphPad Prism version 11 (GraphPad Software, San Diego, USA). Descriptive statistics were calculated using measures of absolute and relative frequency, mean, median, interquartile range (IQR), and standard deviation (± SD). The chi-square test or Fisher’s exact test was used to compare the frequencies of categorical variables between groups. The normality of the sample distribution was assessed using the Shapiro-Wilk test. After this processing, the Mann-Whitney test was used to compare salivary cytokine levels between categories. Spearman’s correlation coefficient (rho) was estimated to analyze the correlation between measures.

Receiver operating characteristic (ROC) curve analysis was performed to evaluate the ability of salivary cytokine levels to identify patients with acromegaly and estimate the area under the curve (AUC), sensibility, and specificity. The optimal cutoff point for identifying acromegaly was then determined using the Youden index.

Furthermore, principal component regression (PCR) was performed to evaluate the joint contribution of the salivary cytokines and potential confounding variables to acromegaly status. Acromegaly status was considered the dependent variable, with participants classified as either acromegaly or control. The explanatory variables included the salivary concentrations of IL-1β, IL-4, IL-6, IL-10, IL-17 A, IL-22, TGF-α, and TNF-α, together with age, sex, and type 2 diabetes. Since several of the cytokines were correlated, principal component analysis (PCA) was first used to summarize their correlation structure into a smaller number of orthogonal components. Two principal components (PC1 and PC2) were retained for the regression analysis because they explained 47.1% of the variance. The retained principal component scores were subsequently entered into the regression model. The coefficients estimated for the principal components were back-transformed to the original variable scale using the PCA loading matrix, allowing the results to be expressed as beta coefficients for the original cytokines and covariates. The significance level adopted for all analyses was 5% (*p* < 0.05).

## Results

### General characterization

A total of 50 adults were included in the present study (25 with acromegaly and 25 controls). Both groups consisted of 15 women (60%) and 10 men (40%). The mean age was 51.1 ± 15.8 years for cases and 49.1 ± 14.1 years for controls (*p* = 0.632). The prevalence of arterial hypertension, chronic kidney disease, arthrosis, dyslipidemia, and smoking status also did not differ significantly between groups. However, type 2 diabetes had a frequency of 56% in the acromegaly group and no cases in the control group (*p* < 0.001). The mean BMI was 28.2 ± 4.9 kg/m² for cases and 27.8 ± 4.8 for controls (*p* = 0.829). Among patients with acromegaly, 52.0% had biochemical control of IGF-1, with a mean IGF-1 level of 136.0 ± 92.7% of the upper limit of normal (Table 1).

**Table 1:** General characteristics of the study groups

Variables	Acromegaly group		Control group		*p*
Sex [n (%)]					1.000
Female	15	(60.0)	15	(60.0)	
Male	10	(40.0)	10	(40.0)	
Age in years [mean ± SD]	51.1	± 15.8	49.1	± 14.1	0.632
Comorbidities					
Arterial hypertension	12	(48.0)	7	(28.0)	0.145
Type 2 diabetes	14	(56.0)	0	(0)	< 0.001
Chronic kidney disease	1	(4.0)	0	(0)	1.000
Arthrosis	4	(16.0)	2	(8.0)	0.667
Dyslipidemia	8	(32.0)	7	(28.0)	0.758
Smoking status [n, (%)]					0.702
Non-smoker	20	(80.0)	22	(88.0)	
Former smoker	5	(20.0)	3	(12.0)	
BMI in kg/m² [mean ± SD]	28.2	± 4.9	27.8	± 4.8	0.829
IGF-1 biochemical control [n (%)]	13	(52.0)	—	—	
% ULN [mean ± SD]	136.0	92.7			
Fasting glucose in mg/dL [mean ± SD]	120	± 48.9	—	—	
HbA1c in % [mean ± SD]	6.8	± 1.7	—	—	

Abbreviations: *±SD* standard deviation, *BMI* body mass index, *IGF-1* insulin-like growth factor-1, *% ULN* percentage of the upper limit of the normal range of IGF-1, *HbA1c*, glycated hemoglobin

### Salivary cytokine profile in acromegaly

**Fig. 1:**
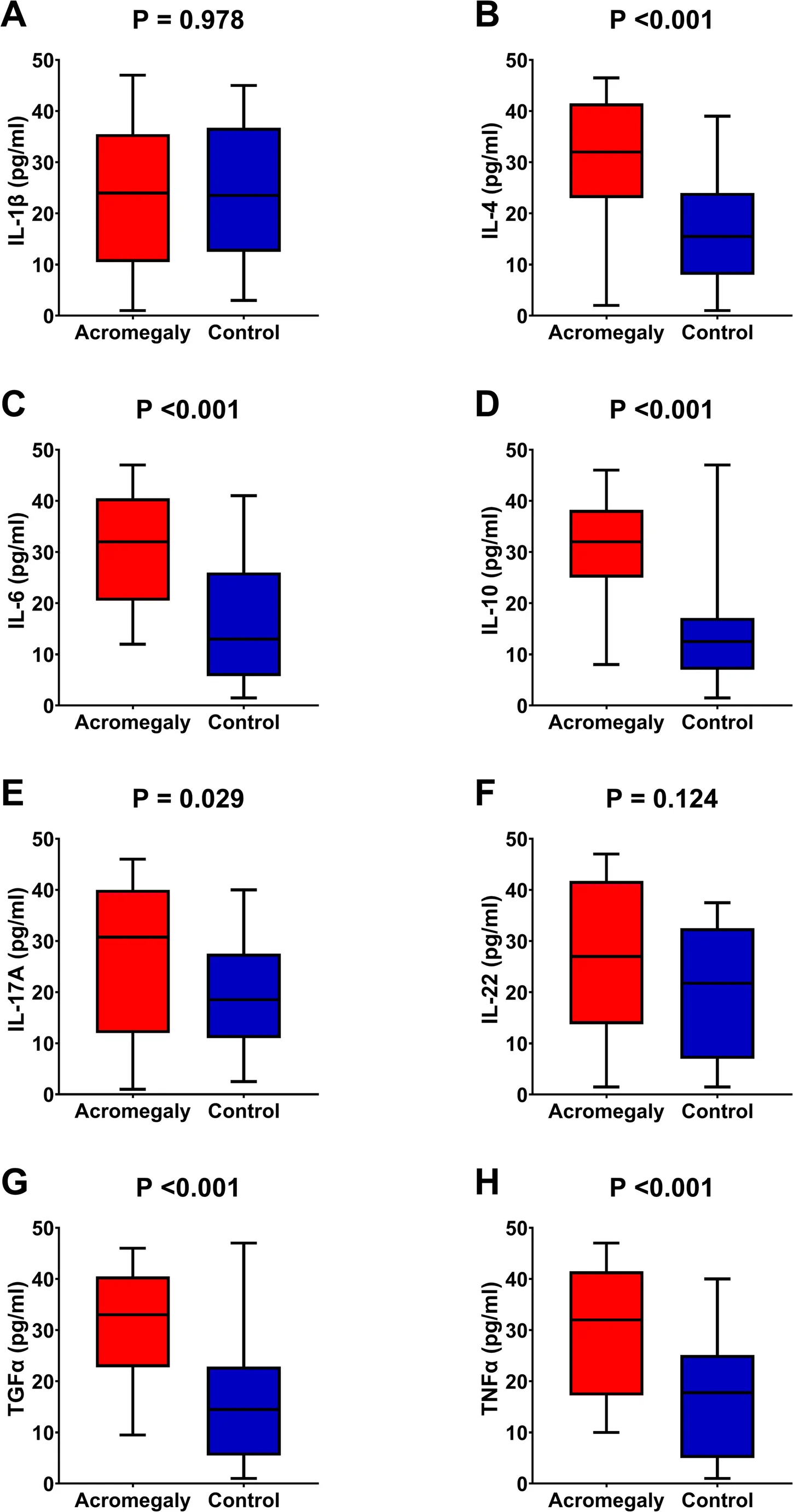
Comparative analysis of salivary levels of IL-1β (**A**), IL-4 (**B**), IL-6 (**C**), IL-10 (**D**), IL-17 A (**E**), IL-22 (**F**), TGF-α (**G**), and TNF-α (**H**). The box plots show the full range (whiskers) and the 1st, 2nd, and 3rd quartiles (box) of the descriptive statistics for each group. Abbreviations: *IL*, interleukin; *TGF*, transforming growth factor; *TNF*, tumor necrosis factor

Figure 1 presents the comparative analysis of salivary levels of pro- and anti-inflammatory cytokines. The acromegaly group had higher levels than controls for the pro-inflammatory cytokines IL-6 (*p* < 0.001), IL-17 A (*p* = 0.029), TGF-α (*p* < 0.001), and TNF-α (*p* < 0.001). Higher levels were also detected for the anti-inflammatory cytokines IL-4 (*p* < 0.001) and IL-10 (*p* < 0.001) in the acromegaly group. No significant differences were observed between the groups for isolated IL-1β and IL-22.

ROC curve analyses (Table 2 and Supplementary Fig. 1) indicate that the strongest signatures for acromegaly classification were observed for IL-10 (AUC = 0.832, cutoff > 1.04 pg/mL, sensitivity = 96% and specificity = 77.3%), TGF-α (AUC = 0.821, cutoff > 16.2 pg/mL, sensitivity = 76%, specificity = 77.3%), IL-4 (AUC = 0.793, cutoff > 7.82 pg/mL, sensitivity = 72%, specificity = 86.4%), IL-6 (AUC = 0.792, cutoff > 4.23, sensitivity = 92%, specificity = 54.5%), and TNF-α (AUC = 0.780, cutoff > 74.8 pg/mL, sensitivity = 64%, specificity = 81.8%).

**Table 2:** Parameters of the ROC curve analysis for the classification of acromegaly based on salivary cytokine levels

Cytokines	Cutoff	Sensitivity	95% CI	Specificity	95% CI	AUC	IC95%
IL-1β	< 15.6	24.0%	11.5%–43.4%	86.4%	66.7%–95.3%	0.503	0.335–0.670
IL-4	> 7.82	72.0%	52.4%–85.7%	86.4%	66.7%–95.3%	0.793	0.660–0.926
IL-6	> 4.23	92.0%	75.0%–98.6%	54.5%	34.7%–73.1%	0.792	0.662–0.922
IL-10	> 1.04	96.0%	80.5%–99.8%	77.3%	56.6%–89.9%	0.832	0.693–0.970
IL-17 A	> 0.25	50.0%	31.4%–68.6%	86.4%	66.7%–95.3%	0.686	0.526–0.845
IL-22	> 240	36.0%	20.2%–55.5%	100%	85.1%–100%	0.632	0.472–0.792
TGF-α	> 16.2	76.0%	56.6%–88.5%	77.3%	56.6%–89.9%	0.821	0.696–0.946
TNF-α	> 74.8	64.0%	44.5%–79.8%	81.8%	61.5%–92.7%	0.780	0.649–0.911

Abbreviations: *ROC* Receiver Operating Characteristic, *95% CI *95% confidence interval, *AUC* Area under the ROC curve, *IL* interleukin, *TGF* transforming growth factor, *TNF *tumor necrosis factor

### Ratio of pro-/anti-inflammatory cytokines

Comparative analysis showed significant differences in the IL-22/IL-4 (*p* < 0.001), IL-1β/IL-10 (*p* = 0.023), and IL-22/IL-10 (*p* = 0.003) ratios, all of which were lower in adults with acromegaly than in controls (Table 3). Because IL-1β and IL-22 did not differ significantly between groups, whereas IL-4 and IL-10 were significantly higher in the acromegaly group, the lower ratios appear to be primarily driven by increased concentrations of the anti-inflammatory cytokines used as denominators. Therefore, these ratios should be interpreted as reflecting an altered relative cytokine balance rather than as evidence of a more pro-inflammatory profile in acromegaly.

**Table 3:** Comparative analysis of the ratios of pro- to anti-inflammatory cytokines between the acromegaly and control groups

Ratio	Acromegaly group		Control group		Effect size	*p*
	Med	IQR	Med	IQR		
IL-1β/IL-4	9.72	19.1	12.8	34.9	0.287	0.094
IL-6/IL-4	1.69	4.85	1.17	2.18	0.243	0.158
IL-17 A/IL-4	0.01	0.15	0.02	0.02	0.068	0.703
IL-22/IL-4	9.64	9.39	30.7	36.5	0.614	< 0.001
TGF-α/IL-4	1.72	4.4	2.19	3.77	0.116	0.505
TNF-α/IL-4	8.49	9.63	9.87	17.3	0.156	0.368
IL-1β/IL-10	22.2	48.1	46.5	152	0.385	0.023
IL-6/IL-10	5.61	5.51	2.1	6.88	0.298	0.082
IL-17 A/IL-10	0.08	0.11	0.11	0.11	0.077	0.660
IL-22/IL-10	37.7	52.2	140	144	0.494	0.003
TGF-α/IL-10	6.71	6.99	7.78	8.59	0.094	0.587
TNF-α/IL-10	20.6	28.9	41.2	53.1	0.163	0.343

Abbreviations: *Med* median, *IQR* interquartile range, *IL* interleukin, *TGF* transforming growth factor, *TNF* tumor necrosis factor

### Correlation between salivary cytokine levels

**Fig. 2:**
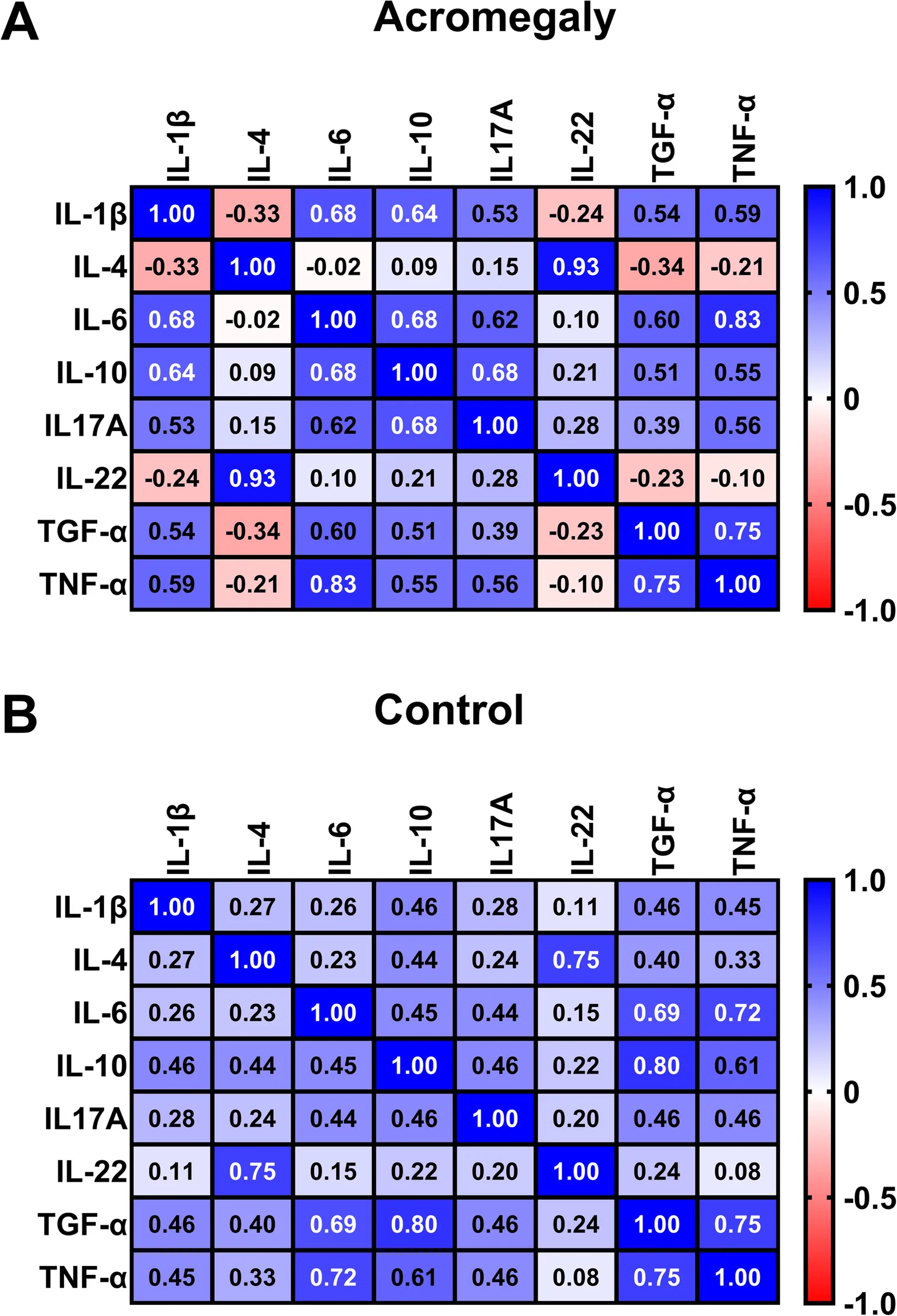
Heat map showing the Spearman correlation coefficients for salivary cytokine levels in the acromegaly (**A**) and control (**B**) groups. Abbreviations: *IL*, interleukin; *TGF*, transforming growth factor; *TNF*, tumor necrosis factor

Figure 2 and Supplementary Table 1 present the correlation analysis between cytokines in each group. Most correlation patterns were similar between the groups, as observed in the correlations of TNF-α with other cytokines. The strongest direct correlations in the acromegaly group were between IL-4 and IL-22 (Rho = 0.933, *p* < 0.001) and between IL-6 and TNF-α (Rho = 0.826, *p* < 0.001). Differences in the acromegaly group were identified for the direct correlation between IL-1β and IL-6 (Rho = 0.675, *p* < 0.001), IL-1β and IL-17 A (Rho = 0.528, *p* = 0.008), IL-1β and TGF-α (Rho = 0.542, *p* = 0.005), and TGF-α and IL-17 A (Rho = 0.463, *p* = 0.030). The significant correlation between IL-4 and IL-10 was not identified in the acromegaly group.

### Salivary cytokine levels and disease activity

A comparative analysis was performed to investigate whether salivary cytokine levels were associated with disease activity and metabolic control in the acromegaly group (Table 4). Regarding IGF-1 control, a higher salivary IL-6 levels were observed in uncontrolled patients (*p* = 0.048). The IL-6/IL-4 and IL-6/IL-10 ratios were significantly higher in the uncontrolled group (*p* = 0.022 and *p* = 0.030, respectively). When stratifying by glycemic control, patients with HbA1c ≥ 7% exhibited significantly higher levels of IL-6 (*p* = 0.028) and TNF-α (*p* = 0.043), as well as an elevated IL-6/IL-4 ratio (*p* = 0.019), compared to those with HbA1c < 7%. Similarly, patients with fasting glucose ≥ 110 mg/dL presented higher levels of IL-1β (*p* = 0.043) and lower levels of IL-4 (*p* = 0.030) and IL-22 (*p* = 0.033) compared to those with normoglycemia. Furthermore, the ratios of IL-1β/IL-4 (*p* = 0.005), IL-6/IL-4 (*p* = 0.004), TGF-α/IL-4 (*p* = 0.010), TNF-α/IL-4 (*p* = 0.002), and IL-1β/IL-10 (*p* = 0.039) were all significantly elevated in patients with higher fasting glucose levels.

**Table 4:** Comparative analysis of salivary cytokine levels according to IGF-1 biochemical control. serum HbA1c. and fasting glucose levels in the acromegaly group

Variables	IGF-1 biochemical control				*p*	HbA1c ≥ 7%				*p*	Fasting glucose ≥ 110 mg/dL				*p*
	Controlled		Uncontrolled			No		Yes			No		Yes		
	Med	IQR	Med	IQR		Med	IQR	Med	IQR		Med	IQR	Med	IQR	
IL-1β	46.5	182.0	84.6	311.0	0.446	49.6	160.0	201.0	674.0	0.198	32.9	84.2	245.0	1135.0	0.043
IL-4	21.1	33.3	12.3	19.9	0.327	19.2	34.5	17.1	13.7	0.619	29.5	32.5	11.5	18.2	0.030
IL-6	12.4	17.9	39.6	64.1	0.048	11.6	16.9	33.4	50.7	0.028	15.1	22.5	24.2	42.5	0.184
IL-10	2.43	4.68	4.2	5.42	0.430	2.59	3.24	5.94	9.07	0.177	2.59	2.98	5.94	5.27	0.355
IL-17 A	0.17	0.59	0.31	1.22	0.140	0.19	0.67	0.40	0.97	0.419	0.19	0.19	0.61	1.05	0.537
IL-22	198.0	213.0	163.0	158.0	0.586	187.0	192.0	162.0	190.0	0.770	240.0	121.0	95.7	78.2	0.033
TGF-α	20.3	14.6	27.6	20.9	0.341	19.1	14.2	25.8	25.6	0.188	19.1	13.4	27.2	24.5	0.141
TNF-α	76.2	75.8	125.0	152.0	0.109	73.0	77.6	133.0	122.0	0.043	64.1	68.5	120.0	93.6	0.053
IL-1β/IL-4	1.71	30.60	9.97	11.10	0.503	5.40	13.70	9.85	23.70	0.259	0.98	9.24	11.90	140.00	0.005
IL-6/IL-4	0.77	2.13	4.89	5.12	0.022	0.77	2.14	5.54	6.02	0.019	0.59	1.48	5.16	5.12	0.004
IL-17 A/IL-4	0.01	0.03	0.09	0.17	0.089	0.01	0.15	0.05	0.18	0.257	0.01	0.02	0.09	0.17	0.118
IL-22/IL-4	8.09	6.48	11.5	9.30	0.110	8.41	6.30	10.20	7.19	0.472	7.98	6.05	11.10	9.47	0.089
TGF-α/IL-4	0.87	1.46	2.23	4.75	0.123	1.29	2.00	1.70	3.72	0.212	0.64	1.57	3.00	7.35	0.010
TNF-α/IL-4	3.94	11.20	10.10	10.50	0.098	7.59	11.10	8.40	5.31	0.312	3.99	7.51	12.40	26.50	0.002
IL-1β/IL-10	28.4	51.1	18.7	31.2	0.936	17.9	34.0	28.0	105.0	0.585	6.4	20.3	43.9	126.0	0.039
IL-6/IL-10	4.29	3.60	6.99	8.34	0.030	3.34	4.00	6.20	3.29	0.084	3.60	4.48	5.79	3.54	0.242
IL-17 A/IL-10	0.07	0.07	0.10	0.25	0.143	0.08	0.14	0.08	0.10	0.879	0.06	0.08	0.09	0.13	0.413
IL-22/IL-10	67.2	113.0	29.9	18.3	0.347	59.2	94.6	26.8	24.6	0.192	61.6	92.4	21.6	28.5	0.060
TGF-α/IL-10	6.71	6.64	6.66	5.53	0.810	6.61	3.17	6.54	10.80	0.977	6.30	4.33	7.37	5.94	0.514
TNF-α/IL-10	20.0	32.4	26.00	25.20	0.568	19.5	22.9	24.4	33.4	0.578	19.5	9.9	33.0	36.3	0.371

Abbreviations: *IGF-1* insulin-like growth factor-1, *HbA1c* glycated hemoglobin, *Med* median, *IQR* interquartile range, *IL* interleukin, *TGF* transforming growth factor, *TNF* tumor necrosis factor

### Multivariate PCA analysis

Supplementary Fig. 2 presents the distribution of individual PC scores and the corresponding variable loadings, PC1 and PC2 explained 47.1% of the variance. After back-transformation of the component coefficients to the original variable scale, IL-4 (*p* = 0.009), IL-6 (*p* = 0.001), IL-17 A (*p* = 0.001), IL-22 (*p* = 0.005), and TNF-α (*p* = 0.021) remained significantly associated with acromegaly status, even considering the effects of confounders (Table 5).

**Table 5:** Back-transformed beta coefficients from the principal component regression model for acromegaly status

Factors	Beta	SE	t	*P* value
Intercept	0.2313	0.1206	1.917	0.061
IL-1β	0.0000	0.0000	1.289	0.204
IL-4	0.0038	0.0014	2.723	0.009
IL-6	0.0010	0.0003	3.329	0.001
IL-10	0.0029	0.0023	1.278	0.208
IL-17 A	0.0049	0.0014	3.527	0.001
IL-22	0.0006	0.0002	2.902	0.005
TGF-α	–0.0001	0.0002	0.328	0.744
TNF-α	0.0004	0.0002	2.391	0.021
Age	0.0004	0.0008	0.547	0.586
Male	–0.0129	0.0376	0.343	0.733
T2D	0.0668	0.0188	3.542	0.001

Abbreviations: *beta* back-transformed regression coefficient, *SE *standard error, *IL* interleukin, *TGF* transforming growth factor, *TNF* tumor necrosis factor, *T2D* type 2 diabetes

## Discussion

To the best of our knowledge, this study provides the first evidence that acromegaly is associated with a distinct salivary cytokine signature. The main findings of this study suggest that acromegaly may be associated with alterations in the levels of pro- and anti-inflammatory cytokines in saliva. Even after adjusting for confounding variables, we found that acromegaly was associated with increased levels of the pro-inflammatory cytokines IL-6, IL-17, IL-22 and TNF-α, as well as increased levels of the anti-inflammatory cytokine IL-4.

An elevated profile of pro-inflammatory cytokines can have important biological consequences, particularly when accompanied by alterations in the relative balance among inflammatory mediators [20]. In the present study, however, the cytokine ratio findings require a cautious interpretation. The IL-22/IL-4, IL-1β/IL-10, and IL-22/IL-10 ratios were significantly lower in patients with acromegaly than in controls. Because IL-1β and IL-22 did not differ significantly between groups, whereas IL-4 and IL-10 were significantly increased in patients with acromegaly, the lower ratios appear to be mainly attributable to increased levels of the anti-inflammatory cytokines used as denominators. Thus, these ratios indicate an alteration in the relative proportions of inflammatory mediators, particularly involving increased IL-4 and IL-10.

In acromegaly, it is well documented that patients present a state of systemic inflammation, more prevalent in patients with active disease, but still present in treated patients [21]. In the present study, the stratification of patients with acromegaly according to disease activity revealed elevated salivary IL-6 levels, IL-6/IL-4, and IL-6/IL-10 ratios in patients with uncontrolled disease suggests that active acromegaly may promote a relative pro-inflammatory shift, even when other cytokine differences do not reach statistical significance. These findings suggest that salivary IL-6 and its ratios with anti-inflammatory cytokines may serve as sensitive indicators of disease activity.

The present findings show that, despite the presence of elevated anti-inflammatory cytokines compared to controls, the pattern of pro-/anti-inflammatory ratios indicates an activation of the anti-inflammatory pathway, suggesting a compensatory mechanism to mitigate the effects of exacerbated pro-inflammatory cytokine production. Among the cytokines elevated in the saliva of patients with acromegaly, IL-6 stands out, a pro-inflammatory cytokine that, when at high levels, can promote an exacerbated inflammatory response. This phenomenon is concerning, as chronic inflammation is associated with several pathological conditions [22]. The concentration of IL-6 in patients with acromegaly remains debated. Some studies have shown higher serum IL-6 levels in acromegaly patients than in controls [23, 24], while others have not found significant differences between the two groups [25, 26]. However, these previous studies have only analyzed serum IL-6 levels.

Another cytokine with elevated levels in the saliva of patients with acromegaly in this study was IL-17. IL-17 exerts pro-inflammatory effects, especially when associated with other cytokines such as TNF-α, IL-1β, and IL-22, and plays a central role in the immune response, connecting innate and adaptive responses [27, 28]. IL-17 and IL-22 have synergistic effects, although their functions may diverge: while IL-17 is essential for defense against infections, IL-22 contributes to homeostasis and may also have pro-inflammatory effects during inflammation [29]. The imbalance between these two cytokines may contribute to chronic inflammation in diseases such as arthritis and psoriasis [30–31]. The salivary increase in IL-17 and IL-22 observed in this study, combined with significant differences in the ratios with IL-10, may suggest that acromegaly contributes to the dysregulation of the interaction between these inflammatory mediators, which could generate abnormal inflammatory reactions in this group.

Regarding TNF-α, an important cytokine for regulating the inflammatory response in several autoimmune diseases [32]. Previous studies have found higher serum TNF-α levels in patients with active acromegaly than in controls [23, 25]. The present result suggest that this increase can also be found in saliva. These findings suggest an association between acromegaly and the systemic inflammatory state of these patients. The increase could be a compensatory response to chronic exposure to excess GH in hepatocytes [25].

The elevation of anti-inflammatory cytokines, particularly IL-4 and IL-10, in the acromegaly group is an interesting finding. This may indicate a compensatory immunoregulatory mechanism aimed at counteracting the effects of elevated pro-inflammatory cytokine production [21]. These results suggest an enhanced regulatory response to elevated levels of pro-inflammatory cytokines, including IL-6, IL-17 A, TGF-α, and TNF-α. Therefore, the salivary cytokine profile observed in acromegaly appears to involve simultaneous changes in pro- and anti-inflammatory mediators rather than a unidirectional shift toward a pro-inflammatory state. Further studies are needed to determine whether these alterations reflect compensatory mechanisms, disease-related immune dysregulation, or metabolic influences.

The anti-inflammatory cytokine IL-4, which plays a role in inhibiting pro-inflammatory cytokines such as IL-1, IL-6, IL-10, IL-12, and TNF-α [33], was also found at elevated levels in the saliva of patients with acromegaly in the present study. IL-4 counterbalances the inflammatory response, a role well documented in diseases such as psoriasis [34] and type 1 diabetes [35], and may also be associated with inflammatory arthritis [36]. These findings reinforce the hypothesis that IL-4 may exert a regulatory effect on the immune response of these patients. The imbalance between pro- and anti-inflammatory cytokines observed in this study, evidenced by the ratios between them, suggests a pro-inflammatory profile in the saliva of patients with acromegaly. Such dysregulation may compromise the immune response and may be associated with an increased risk of comorbidities, including cardiovascular and liver diseases and endothelial alterations, in addition to contributing to increased mortality in acromegaly [37–39].

The stratification of patients with acromegaly according to metabolic control revealed important associations between salivary cytokine profiles and glycemic status. Patients with poorer glycemic control exhibited higher levels of IL-6, TNF-α, and IL-1β, as well as lower levels of IL-4, and several pro-/anti-inflammatory cytokine ratios were also altered according to fasting glucose and HbA1c categories. These findings indicate that metabolic status may contribute substantially to the salivary inflammatory profile observed in this population. These findings are clinically relevant, given that diabetes is a common comorbidity in acromegaly [40], and is associated with increased cardiovascular risk and mortality [41]. Elevated pro-to-anti-inflammatory cytokine ratios, particularly IL-6/IL-4 and TNF-α/IL-4, in hyperglycemic patients further support the notion that metabolic disturbances can exacerbate the inflammatory burden in acromegaly. This is consistent with previous reports demonstrating that hyperglycemia promotes a pro-inflammatory state through the activation of the nuclear factor kappa-B (NF-κB) pathway and increased production of reactive oxygen species [42]. Furthermore, the present results suggest that salivary cytokines may serve as accessible biomarkers for monitoring not only acromegaly-related inflammation but also the metabolic complications associated with the disease.

A strength of the present study was the statistical strategy adopted, using multivariate techniques. This approach allowed us to evaluate possible interactions between inflammatory molecules, better mimicking the biological behavior of these factors. The principal component analysis reduced the dimensionality of the correlated variables, and the resulting component scores were incorporated into the regression model. The back-transformed coefficients indicated that IL-4, IL-6, IL-17 A, IL-22, and TNF-α contributed significantly to the multivariate association with acromegaly status, whereas age and sex did not show statistically significant associations. This indicates that the observed salivary cytokine alterations are not merely a consequence of glycemic status but can be related to acromegaly itself. However, considering inflammation as a complex process, new studies should include novel molecules that could broaden the understanding of the mechanisms involved in the relationship between acromegaly and inflammatory alterations. Additionally, the use of saliva as a biological matrix represents a significant methodological advantage, as it enables non-invasive, repeatable sampling with minimal patient discomfort and lower risk of infection compared to blood collection.

A limitation of the present study was the small sample size. However, this limitation was mitigated by matching controls for demographic factors. Therefore, we recommend that future studies adopt longitudinal designs and include larger samples to allow for causal inference of the investigated factors. Additionally, diabetes was an exclusion criterion for the control group. This resulted in limited covariate overlap, restricting the ability of multivariate adjustment to fully control for confounding factors related to diabetes. Therefore, the present findings should be considered hypothesis-generating rather than definitive evidence that the observed cytokine alterations are solely attributable to acromegaly. Studies that include both diabetic and non-diabetic participants in the acromegaly and control groups are necessary to more clearly distinguish the effects of the disease from metabolic effects. Another limitation of the present study is that the analysis did not consider treatment protocols or medication use. The pharmacological treatment of acromegaly and medications used to treat associated comorbidities may influence inflammatory pathways and cytokine concentrations. Future studies should incorporate detailed medication histories and treatment characteristics to investigate their potential impact on salivary cytokine concentrations.

Given the imbalanced cytokine signature observed in this study, further studies should be conducted to investigate the relationship between these findings and the development of inflammatory conditions and possible tissue damage in oral structures and distant organs. These data may support the feasibility of using saliva as a biomarker to monitor the inflammatory status of patients with acromegaly.

## Conclusion

The findings of this study suggest that patients with acromegaly exhibit an altered salivary cytokine profile involving both pro- and anti-inflammatory mediators. Significant increases were observed in IL-4, IL-6, IL-10, IL-17 A, TGF-α, and TNF-α, whereas IL-1β and IL-22 did not differ significantly between groups. The IL-22/IL-4, IL-1β/IL-10, and IL-22/IL-10 ratios were lower in acromegaly, apparently reflecting the higher levels of IL-4 and IL-10 rather than an increase in the relative pro-inflammatory components. Multivariate analysis showed that several cytokines remained statistically associated with acromegaly after inclusion of age, sex, and type 2 diabetes. Further studies with larger samples and adequate representation of diabetic and non-diabetic participants in both groups are needed to clarify the respective contributions of acromegaly and metabolic status to salivary cytokine alterations.

## Supplementary Material

Below is the link to the electronic supplementary material.

Supplementary Material 1

## Data Availability

The datasets generated and/or analyzed during the current study are available from the corresponding author on reason¬able request.
