# Supplementary material for: Pro- and anti-inflammatory cytokine dysregulation in the saliva of patients with acromegaly: a cross-sectional study
Source: Pituitary. 2026 Oct 16;29(6):166. doi: 10.1007/s11102-026-01767-6 (PMC13675317; doi:10.1007/s11102-026-01767-6)
Supplement: Supplementary file 1 [file 11102_2026_1767_MOESM1_ESM.docx]

Supplementary Fig. 1. Receiver Operating Characteristic (ROC) curve analysis for the classification of acromegaly based on salivary levels of IL-1β (A), IL-4 (B), IL-6 (C), IL-10 (D), IL-17A (E), IL-22 (F), TGF-α (G), and TNF-α (H). Abbreviations: IL, interleukin; TGF, transforming growth factor; TNF, tumor necrosis factor.
Supplementary Table 1. Spearman’s correlation matrix for salivary cytokine levels in the acromegaly and control groups.
Variables
IL-1β
IL-4
IL-6
IL-10
IL-17A
IL-22
TGF-α
Rho
p
Rho
p 
Rho
p
Rho
p 
Rho
p
Rho
p
Rho
p 
Acromegaly group


IL-4
-0.327
—


0.111
—


IL-6
0.675
-0.024
—


<0.001
0.908
—


IL-10
0.635
0.094
0.676
—


<0.001
0.656
<0.001
—


IL-17A
0.528
0.147
0.617
0.681
—


0.008
0.493
0.001
<0.001
—


IL-22
-0.244
0.933
0.097
0.215
0.282
—


0.240
<0.001
0.646
0.302
0.183
—

TGF-α
0.542
-0.343
0.605
0.513
0.387
-0.228
—

0.005
0.094
0.001
0.009
0.062
0.274
—
TNF-α
0.588
-0.214
0.826
0.550
0.560
-0.100
0.752

0.002
0.304
<0.001
0.004
0.004
0.633
<0.001
Control group


IL-4
0.269
—


0.225
—


IL-6
0.257
0.225
—


0.248
0.314
—


IL-10
0.463
0.441
0.451
—


0.030
0.040
0.035
—


IL-17
0.279
0.244
0.443
0.462
—


0.209
0.273
0.039
0.030
—


IL-22
0.113
0.750
0.146
0.220
0.205
—


0.616
<0.001
0.518
0.325
0.360
—

TGF-α
0.462
0.397
0.694
0.801
0.463
0.238
—

0.030
0.067
<0.001
<0.001
0.030
0.286
—
TNF-α
0.449
0.329
0.724
0.608
0.456
0.085
0.754

0.036
0.135
<0.001
0.003
0.033
0.707
<0.001
Abbreviations: Rho, Spearman's correlation coefficient; IL, interleukin; TGF, transforming growth factor; TNF, tumor necrosis factor.

Supplementary Fig. 2. Principal component analysis of salivary cytokines and covariates. (A) Distribution of individual scores according to PC1 and PC2, with participants classified according to acromegaly status (A). Biplot showing the loadings of the eight salivary cytokines, age, sex, and type 2 diabetes on PC1 and PC2. The two retained principal components were subsequently used in the principal component regression model (B). Abbreviations: IL, interleukin; TGF, transforming growth factor; TNF, tumor necrosis factor; T2D, type 2 diabetes.
